# The risk factors and predictive modeling of mortality in patients with mental disorders combined with severe pneumonia

**DOI:** 10.3389/fpsyt.2023.1300740

**Published:** 2024-01-11

**Authors:** Yaolin Li, Weiguo Zhou, Huiqin Wang, Jing Yang, Xiayahu Li

**Affiliations:** ^1^Department of Respiratory and Critical Care Medicine, The Third People's Hospital of Chengdu, Affiliated Hospital of Southwest Jiaotong University, Chengdu, China; ^2^Department of Critical Care Medicine, Chengdu Fourth People's Hospital, Chengdu, China; ^3^The Affiliated Women's and Children's Hospital, School of Medicine, UESTC, Chengdu, China; ^4^Department of Critical Care Medicine, Chengdu Second's People Hospital, Chengdu, China

**Keywords:** severe pneumonia, risk factors, predictive modeling, mental disorder, The Montreal Cognitive Assessment

## Abstract

**Background:**

We explored clinical characteristics and risk factors for mortality in patients with mental disorders combined with severe pneumonia and developed predictive models.

**Methods:**

We retrospectively analyzed the data of 161 patients with mental disorders combined with severe pneumonia in the intensive care unit (ICU) of a psychiatric hospital from May 2020 to February 2023, and divided them into two groups according to whether they died or not, and analyzed their basic characteristics, laboratory results and treatments, etc. We analyzed the risk factors of patients' deaths using logistics regression, established a prediction model, and drew a dynamic nomogram based on the results of the regression analysis. Based on the results of regression analysis, a prediction model was established and a dynamic nomogram was drawn.

**Results:**

The non-survivor group and the survivor group of patients with mental disorders combined with severe pneumonia were statistically different in terms of age, type of primary mental illness, whether or not they were intubated, whether or not they had been bedridden for a long period in the past, and the Montreal Cognitive Assessment (MoCA) scale, procalcitonin (PCT), albumin (ALB), hemoglobin (Hb), etc. Logistics regression analysis revealed the following: MoCA scale (OR = 0.932, 95% CI:0.872–0.997), age (OR = 1.077, 95%CI:1.029–1.128), PCT (OR = 1.078, 95% CI:10.006–10.155), ALB (OR = 0.971, 95%CI:0.893–1.056), Hb (OR = 0.971, 95% CI: 0.942–0.986) were statistically significant. The ROC curve showed that the model predicted patient death with an area under the curve (AUC) of 0.827 with a sensitivity of 73.4% and a specificity of 80.4%.

**Conclusion:**

Low MoCA score, age, PCT, and low Hb are independent risk factors for death in patients with mental disorders with severe pneumonia, and the prediction model constructed using these factors showed good predictive efficacy.

## Introduction

Mental disorder refers to a general term for disturbances in the functional activity of the brain, resulting in varying degrees of impairment in mental activities such as cognition, emotion, behavior, and volition (1). The physical and mental health of people with mental disorders has been an important issue in global public health. According to the World Health Organization (WHO), approximately 450 million people worldwide suffer from various mental disorders, and the quality of life and life expectancy of these patients is generally lower than that of the general population (2).

Severe pneumonia is a serious respiratory disease characterized by severe dyspnea, lung infection, and even respiratory failure, and is one of the leading causes of death worldwide (3). A meta-analysis showed that the pre-diagnosis of mental disorders significantly increased the risk of COVID-19 severity and mortality (4). Their particular physical and psychological states, such as side effects of medication, unhealthy lifestyles, and deficits in knowledge and coping with the disease, may put them at increased risk of death when suffering from severe pneumonia (5). In addition, some studies have found that patients with mental disorders may also have problems with access to and utilization of healthcare services when suffering from severe pneumonia, which may be another important factor in their increased risk of death (6). However, only very few studies have examined the risk factors for death from combined severe pneumonia in patients with mental disorders. Therefore, we conducted an in-depth study on the risk factors for mortality in patients with mental disorders combined with severe pneumonia in an attempt to establish a preventive strategy based on modifiable risk factors that are practically important for reducing pneumonia-related mortality in patients with mental disorders, improving their quality of life and optimizing related healthcare services.

## Materials and methods

### Study design and participants

Retrospectively, we collected data on a total of 161 patients with mental disorders combined with severe pneumonia from May 2020 to February 2023 in the ICU of the Chengdu Fourth People's Hospital. Inclusion criteria: (1) long-term hospitalization or follow-up patients in our hospital; (2) met the diagnosis of mental disorders according to the internationally accepted criteria for classification of mental disorders (ICD-10) and the Diagnostic and Statistical Manual of Mental Disorders, 5th edition (DSM-5) of the United States (7); (3) met the diagnostic criteria for severe pneumonia (8); (4) aged>50 years. Exclusion criteria: (1) persons with other systemic infections in combination; (2) persons taking immunosuppressive drugs for a long period or with immunodeficiency; (3) presence of a history of malignant tumor; (4) missing clinical data; (5) those who refused tracheal intubation and mechanical ventilation; (6) patiens being infected with the COVID-19. This study was a retrospective study without patient intervention, which conformed to the ethical standards for human experimentation and was approved by the Ethics Committee of the Chengdu Fourth People's Hospital.

### Data collection

The population included patients who were hospitalized in our hospital for a long period or who attended regular outpatient clinics, so we were able to collect relevant information by checking information on previous visits. Patient's gender, age, type of mental disorder and duration of illness, type of antipsychotics used, MoCA score within 2 months, antibiotic use within 6 months, and the presence of bed rest for up to 6 months, as well as dysphagia and diabetes, were collected. The first PCT and CRP, as well as Hb, ALB, nutritional support regimens, endotracheal intubation, length of hospital stay, bacterial resistance, and patient survival, were collected when patients were admitted to the ICU.

### Statistical analysis

We used SPSS 26.0 statistical software to process the data. Normally distributed data in quantitative information were expressed as mean±SD, and independent samples *t*-test was selected for analysis. The non-normally distributed measurement data were expressed as median and interquartile ranges (IQRs) and analyzed by the Mann-Whitney U test; the count data were expressed as cases (percentage, %), and comparisons between groups were made by Chi-square statistics or Fisher's exact probability test. Influential factors were analyzed by unifactorial and multifactorial logistics regression models, and variables with *P* < 0.05 were included in binary logistic regression models, while covariance analysis was used to exclude the indicators of covariance and to screen the risk factors of patients' deaths. These factors were used to establish a predictive model for prognosis, and Receiver Operating Characteristic (ROC) curve analysis was performed. The risk prediction model was built with R software (R4.0.3), and a Web-based interactive dynamic nomogram was constructed using SHINY version 0.13.2.26. The Hosmer-Lemeshow test was performed on the predictive model, and *P* > 0.1 indicated that the model had a good fit. Bootstrap was used for internal validation to calculate the C-index, which was used to evaluate the discrimination of the predictive model. We plotted calibration curves and Decision curve analysis (DCA) to evaluate how well the predicted results match the actual results.

## Results

### Univariate analysis of mortality in patients

After screening by exclusion criteria, 161 patients were finally included, 97 (59.5%) patients died after treatment, and 64 patients (40.5%) survived (Table 1). Compared with the survival group, patients in the death group were significantly older on average, with a higher proportion of long-term bedridden and organic mental disorders, and lower MoCA scores (*P* < 0.05). And during hospitalization, the patients in the death group had a large rate of tracheal intubation, in addition to a statistically significant difference in PCT, ALB, and Hb at the time of patient admission (*P* < 0.05).

**Table 1:** Comparison of demographic and clinical characteristics of patients in two groups.

**Characteristic**	**Survival group (n=97)**	**Death group (*n* = 64)**	**χ^2^/F**	***P-value***
Sex (%)			0.204	0.651
Male	64 (66.0)	40 (37.5)		
Female	33 (34.0)	24 (62.5)		
Age (years)	72.6 ± 12.9	81.6 ± 16.9		
Types of antipsychotics (%)			1.376	0.241
≤ 1	50 (51.5)	39 (60.9)		
>1	47 (48.5)	25 (39.1)		
Nutritional support			1.447	0.485
Enteral nutrition	64 (66.0)	47 (73.4)		
Parenteral alimentation	18 (18.6)	11 (17.2)		
United nutrition	15 (15.5)	6 (9.4)		
Primary mental illness			9.058	0.003
Non-organic mental disorders	42 (43.3)	13 (20.3)		
Organic mental disorders	55 (56.7)	51 (79.7)		
Disease time (years)			3.139	0.208
≤ 4	32 (33.0)	24 (37.5)		
4–9	28 (28.9)	24 (37.5)		
≥9	37 (38.1)	16 (25.0)		
Diabetes mellitus			2.492	0.114
No	76 (78.4)	43 (67.2)		
Yes	21 (21.6)	21 (32.8)		
Trachea cannula			12.217	< 0.001
No	65 (67.0)	25 (39.1)		
Yes	32 (33.0)	29 (60.9)		
Long-term bed			20.975	< 0.001
No	66 (68.0)	21 (31.3)		
Yes	31 (32.0)	44 (68.8)		
Dysphagia				
No	55 (56.7)	38 (59.4)	0.113	0.737
Yes	42 (43.3)	26 (40.6)		
Use antimicrobial agents			0.017	0.897
No	49 (50.5)	33 (51.6)		
Yes	48 (49.5)	31 (48.4)		
Bacterial resistance			3.315	0.069
No	49 (50.5)	23 (35.9)		
Yes	48 (49.5)	41 (64.1)		
PCT	0.246 (0.438)	1.179 (4.751)	4.081	< 0.001
CRP	59.110 (105.700)	76.290 (89.250)	1.592	0.111
Length of stay	20 (19)	25 (31)	1.382	0.167
MoCA score	14.804 ± 6.832	11.219 ± 6.586	3.305	0.001
ALB	32.953 ± 5.329	29.391 ± 5.164	4.202	< 0.001
Hb	111.732 ± 19.996	94.906 ± 20.734	5.149	< 0.001

### Binary Logistic regression analysis of mortality in patients

Binary Logistic regression analysis showed that PCT and age were independent risk factors for death in patients with mental disorders combined with severe pneumonia, while MoCA and the levels of Hb were negatively correlated with death in those patients, within certain limits, we can consider low levels of MoCA and Hb as a risk factor for its (Table 2).

**Table 2:** Binary Logistic regression analysis of risk factors.

	**β**	**Standard Error**	**Wald**	**OR (95%CI)**	***P-value***
MoCA	−0.072	0.033	4.882	0.930 (0.872–0.992)	0.027
PCT	0.073	0.034	4.720	1.076 (1.007–1.149)	0.030
ALB	−0.036	0.042	0.471	0.965 (0.888–1.048)	0.394
Hb	−0.030	0.011	7.157	0.970 (0.949–0.992)	0.007
age	0.065	0.022	8.543	1.067 (1.022–1.114)	0.003
Primary mental illness	0.102	0.498	0.042	1.007 (0.417–2.940)	0.838
Trachea cannula	0.756	0.432	3.064	2.129 (0.913–4.961)	0.080
Long-term bed	0.579	0.446	1.686	1.784 (0.745–4.272)	0.579

### Predictive modeling

**Figure 1:**
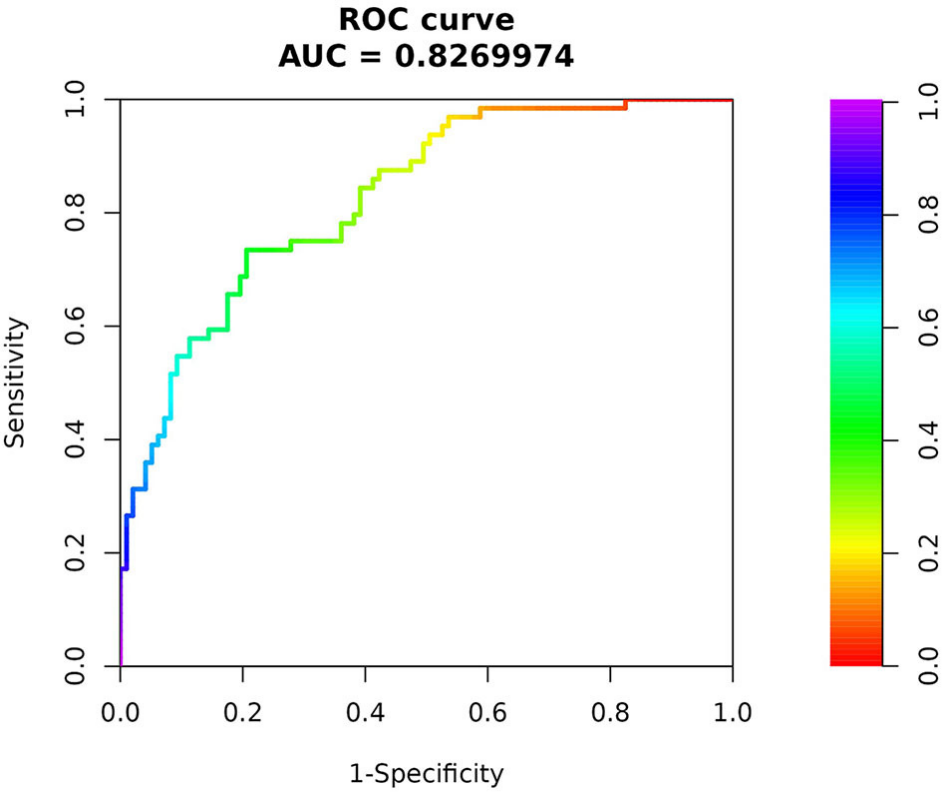
The ROC curve for joint prediction of multiple indicators.

In order to further understand the predictive value of the above indicators for the risk of death in patients with mental disorders combined with severe pneumonia, ROC curves were plotted (Figure 1). The results showed that MoCA score, PCT, Hb and age could be used to predict the risk of death in patients, and showed that when they were used in combination for the prediction of the risk of death in patients, they had the largest area under the curve (AUC) and the best sensitivity and specificity, which was superior to that of the individual variables (Table 3).

**Table 3:** Predictive value of different indicators in ROC curve analysis.

	**Cutoff**	**AUC (95% CI)**	**Sensitivity**	**Specificity**	**Youden index**	***P*-value**
MoCA scores	8.500	0.65 (0.56,0.74)	0.438	0.804	0.242	0.001
PCT	0.493	0.69 (0.61,0.77)	0.672	0.660	0.332	< 0.001
Hb	111.500	0.72 (0.64,0.80)	0.828	0.536	0.364	< 0.001
Age	76.500	0.71 (0.63,0.79)	0.750	0.588	0.338	< 0.001
Joint Indicators	0.460	0.83 (0.76,0.89)	0.734	0.804	0.538	< 0.001

**Figure 2:**
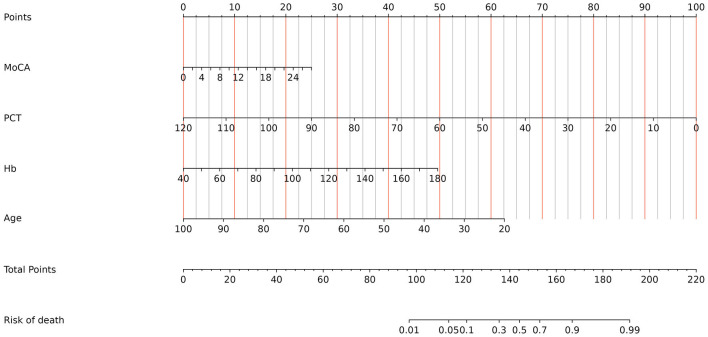
Established nomogram in the cohort by incorporating the following four parameters: age, Hb, MoCA, and PCT.

**Figure 3:**
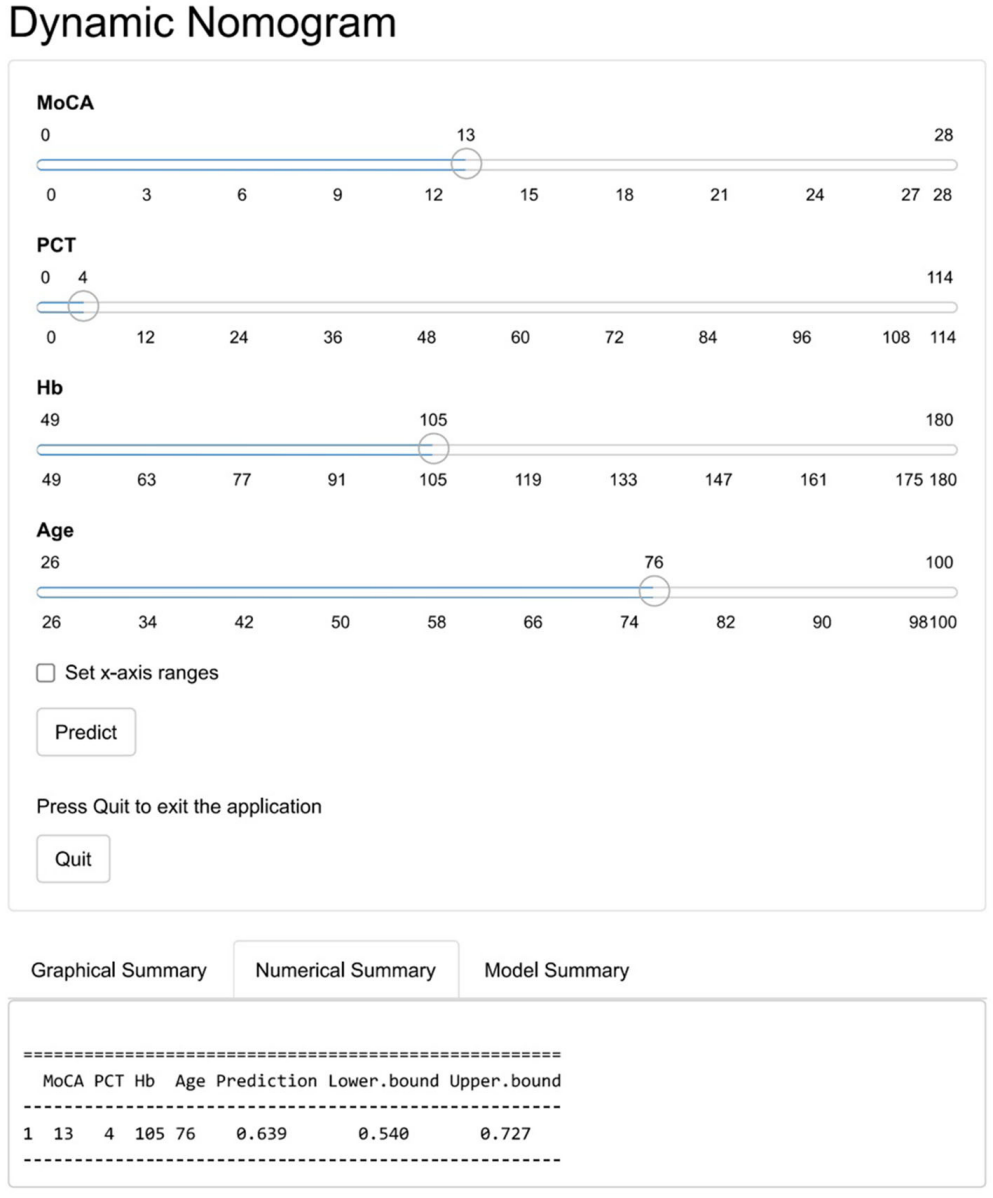
The online dynamic nomogram accessible at: https://jszahbzzfyhzswwxysfx.shinyapps.io/dynnomapp/.

To quantify the above meaningful indicators, an alignment diagram model was drawn by using the R language to obtain the corresponding scores for the different predictors. The scores of all indicators were summed to obtain the total score, which was converted to the corresponding predictive value, i.e., the probability of the risk of death in patients with mental disorders combined with severe pneumonia (Figure 2). To make it easier for clinicians to use our nomogram model, we have also generated an interface that is available on the web (https://jszahbzzfyhzswwxysfx.shinyapps.io/dynnomapp/) (Figure 3).

### Validation of predictive models

**Figure 4:**
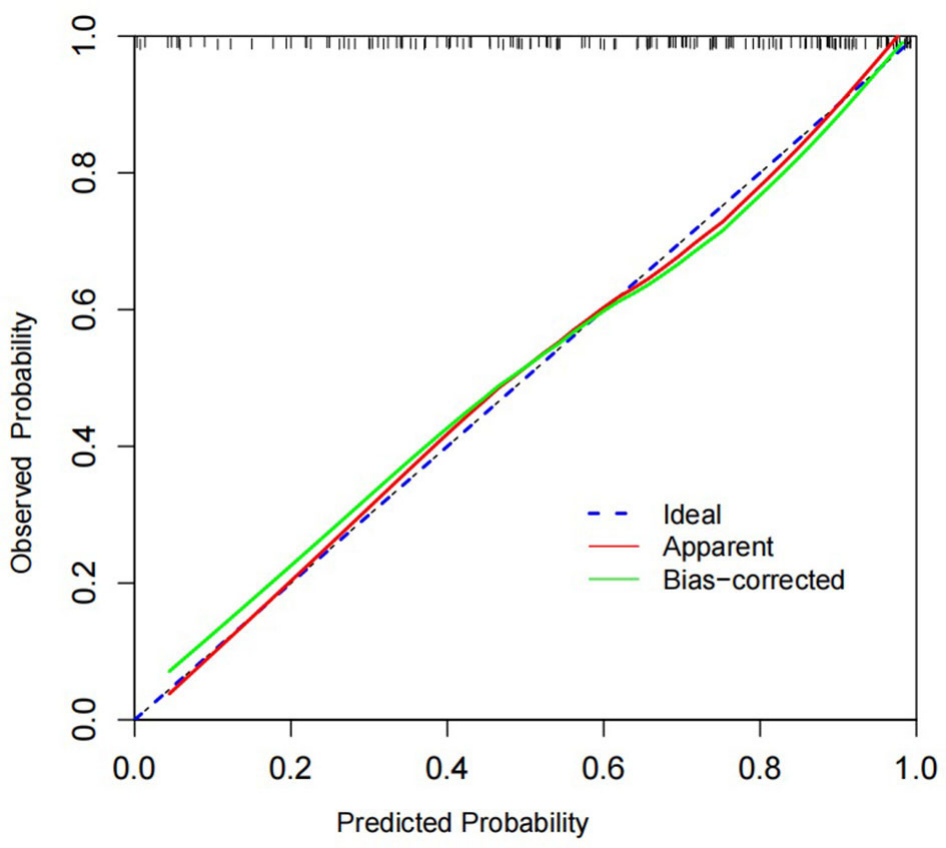
In this figure, the 45° Ideal line is used as a reference standard, and the “apparent” line reflects the fit between the predicted values and the actual values. The Bias-corrected line shows how well the corrected predicted values fit the actual values. We can see that the Bias-corrected line or apparent line is very close to the Ideal line, which indicates a good agreement between the predicted and actual values.

**Figure 5:**
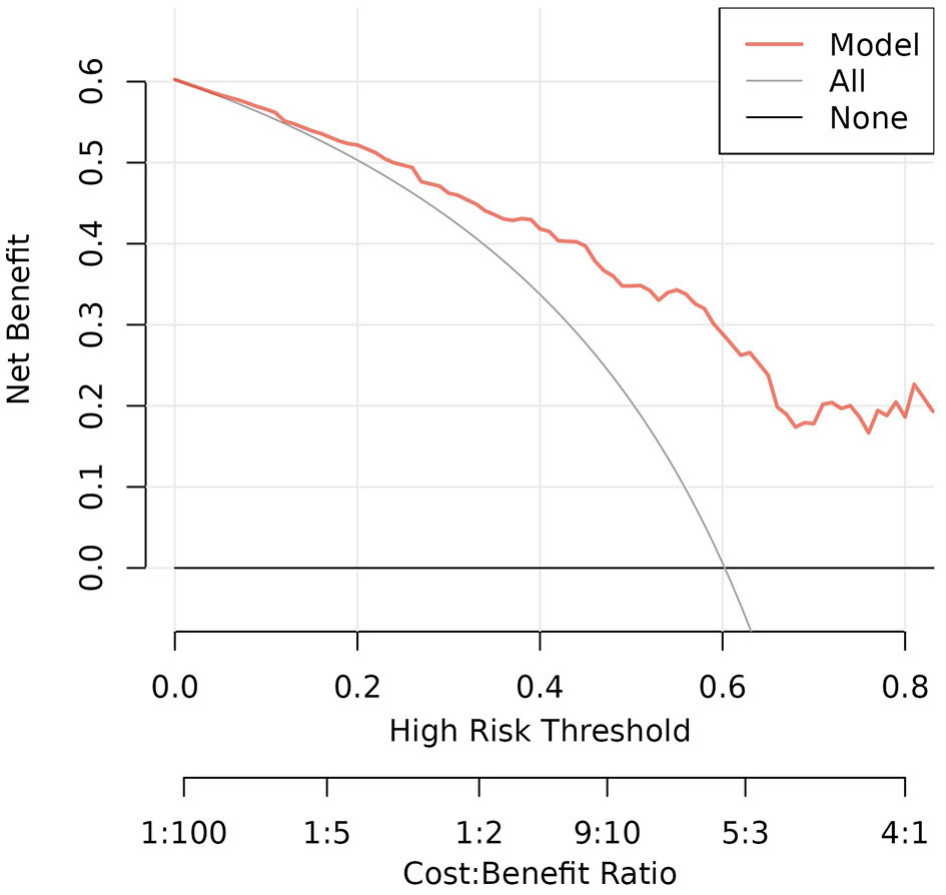
The DCA curve is the one we actually focus on. In the threshold probability range of 0.1 to 0.7, the DCA curve lies above the “None” and “All” baselines, which indicates that the model's performance is acceptable in this range.

To validate the model, Bootstrap was first used to internally validate the model, and the sampling was repeated 1,000 times, and the corrected C-index was obtained to be 0.827, suggesting that the predictive ability was more accurate. At the same time, the calibration curve of the nomogram model was plotted, which was closer to the ideal curve, showing that the model predicted the risk of death in patients with mental disorders combined with severe pneumonia with good consistency with the risk of actual occurrence (Figure 4). The results of the Hosmer-Lemeshow goodness-of-fit test showed that the χ^2^ = 5.549, *P* = 0.698 (*P* > 0.05), indicating a better calibration. The high-risk threshold probability is the probability of a serious deviation in model predictions when clinicians use nomograms for diagnosis and decision-making. In this study, the Decision Curve Analysis (DCA) showed that the nomogram had a good net benefit of clinical use (Figure 5).

## Discussion

In recent years, several studies have shown that patients with mental disorders combined with severe pneumonia have a high in-hospital mortality rate (9), but we have limited knowledge of the risk factors for death in this population. To provide more research information, we systematically analyzed the risk factors for in-hospital mortality in patients with mental disorders combined with severe pneumonia. By analyzing these factors in depth, we can target early intervention and inform future studies.

The results of this study showed a relatively high in-hospital mortality rate of 39.75% in patients with mental disorders combined with severe pneumonia. For the factors related to mental disorders, we collected the type of mental disorders, duration of illness, medication use, recent MoCA scores, and the presence of long-term bedridden. In the univariate comparison between the two groups, we found that the prevalence of organic mental disorders was significantly higher in the death group than in the survival group, and the organic mental disorders in this study mainly consisted of vascular dementia and Alzheimer's disease. This may be due to the prevalence of advanced age, weakened immune response, impairment of the oropharyngeal reflex leading to recurrent aspiration pneumonia in the lungs, and poor lung fundamentals in this group of patients (10, 11). Previous studies have found that antipsychotic medications may increase the risk of developing pneumonia (12–14), but did not indicate their effect on the risk of death after developing pneumonia. And in this study, we did not observe any difference in the type of antipsychotic medication used by the patients for a long time in the two groups. In addition, many older adult patients with psychiatric disorders are chronically bedridden, which may lead to many complications, and pneumonia is one of the most common complications. Bedridden older adult patients, whose basic physiological needs are carried out in bed, are usually accompanied by poor immune, swallowing, and respiratory functions, and therefore have a high risk of developing HAP (15, 16). Due to the combination of multiple complications and the prolonged use of antibiotics and glucocorticoids, they are at increased risk of death once the infection progresses.

PCT is a widely used indicator of inflammation in clinical practice, and its elevated level is associated with the stimulation of multiple inflammatory factors and bacterial toxins. Patients with high levels of PCT tend to have a poor prognosis (17). It has been demonstrated that PCT reflects the severity of severe pneumonia, and decreases as the condition improves (18, 19). Severe pneumonia is mostly caused by bacterial infections, and therefore the level of PCT increases significantly after infection, which is the theoretical basis for PCT as a prognostic predictor for patients with severe pneumonia (20). There is no evidence that PCT is a prognostic indicator for patients with psychiatric disorders combined with severe pneumonia. In the present study, we found that the risk of in-hospital death was significantly increased in patients with high initial PCT values at the time of admission to the ICU [OR = 1.076 (1.007–1.149)], which can be used as an inflammatory indicator and biomarker for assessing the risk of death in such patients.

In this study, it was found that increasing age is an independent risk factor for death in mental disorders combined with severe pneumonia. This may be related to the gradual attenuation of the barrier and immune functions of the respiratory system with increasing age and the decrease in peripheral lymphocytes in the older adult (21, 22). Furthermore, as comorbidities progressively increase with age, the compensatory capacity of other vital organs decreases, and the function of these compromised organs make is difficult to maintain the needs of the organism in the presence of severe infections such as severe pneumonia, and ultimately, multiorgan failure occurs.

The prevalence of anemia observed in psychiatric patients is higher than the average prevalence in the general population, and one survey found that 25.4% of psychiatric patients suffered from anemia, which may be related to the lifestyle, dietary habits, medication use, and underlying physical condition of patients with psychiatric disorders (23–25). In adult community-acquired pneumonia (CAP), the decline in Hb was independently associated with mortality (26–28). Similarly, we found an increase in in-hospital mortality with a decline in Hb in the specific population of mental disorders combined with severe pneumonia. Preventing or treating anemia might improve clinical outcomes in such patients.

The Montreal Cognitive Assessment (MoCA), a 30-item scoring screening tool for assessing a wide range of cognitive declines (29). Originally designed to detect cognitive deficits in older adults with dementia, the MoCA has also been found to be suitable for assessing cognitive functioning in other clinical populations and is now widely used with psychiatric patients (30–32). Dementia is primarily characterized by cognitive dysfunction. During the COVID-19 pandemic, patients with dementia had a higher prevalence of COVID-19 and these patients were also more likely to have more severe presentations and a higher mortality rate due to COVID-19 infections, as well as in patients with depression (33, 34). In this study, the population included contained common psychiatric disorders such as dementia, depression, and schizophrenia, and was older, with the vast majority of the patients experiencing declining levels of cognitive functioning. These patients tend to have poorer health due to cognitive limitations and are often unable to express discomfort correctly, which affects early recognition of pneumonia and leads to delayed intervention for pneumonia.

In addition, the results of our ROC curve analysis showed that the MoCA score had the highest specificity and Hb had the highest sensitivity when a single independent risk factor for death was used to predict the death of these patients and that the maximum AUC and the best sensitivity and specificity were achieved when these indicators were combined. The nomogram model based on the combination of the above multifactors has a better ability to predict the survival of patients. By inputting the corresponding measured values into our online nomogram prediction model, the predicted mortality risk of each patient with mental disorders complicated by severe pneumonia can be clearly displayed, thereby assisting clinicians in early identification of death risk factors and timely implementation of appropriate intervention measures to enhance patients' survival rate.

The research is a single-center retrospective study, and although a rigorous design was adopted, confounding factors may inevitably interfere with and bias the results of the study. For example, neuroimaging was not dynamically evaluated during the treatment diagnosis, which may have influenced the assessment of the patient's condition. Therefore, it is necessary to conduct a multicenter, prospective clinical study on the relevant risk factors and predictive models, as well as rigorous external validation, to further improve the conclusions of this study.

## Conclusion

In summary, this study revealed that cognitive dysfunction, advanced age, PCT, and lower Hb levels were independent risk factors for death in patients with psychiatric disorders combined with severe pneumonia. Predictive models constructed using these factors showed good predictive efficacy. These findings can help to improve clinical decision-making and prognosis in this group of patient.

## Data availability statement

The raw data supporting the conclusions of this article will be made available by the authors, without undue reservation.

## Ethics statement

The studies involving humans were approved by Ethics Committee of Chengdu Fourth People's Hospital. The studies were conducted in accordance with the local legislation and institutional requirements. The ethics committee/institutional review board waived the requirement of written informed consent for participation from the participants or the participants' legal guardians/next of kin because this was a retrospective study, the data came from the medical records system.

## Author contributions

YL: Writing—original draft. WZ: Conceptualization, Writing—review & editing. HW: Investigation, Writing—review & editing. JY: Data curation, Writing—review & editing. XL: Writing—review & editing.
